# 767 Tracking Changes in Pain Ratings between Admission to Discharge at a Regional Burn Center

**DOI:** 10.1093/jbcr/irad045.242

**Published:** 2023-05-15

**Authors:** Lisa Smith, Iman Khan, Tomer Lagziel, Carrie Cox, Julie Caffrey, Sheera Lerman

**Affiliations:** Johns Hopkins Bayview Medical Center, Forest Hill, Maryland; Johns Hopkins University School of Medicine, Baltimore, Maryland; Johns Hopkins Bayview Medical Center, Baltimore, Maryland; Johns Hopkins Adult Burn Center, Baltimore, Maryland; Johns Hopkins University School of Medicine, Baltimore, Maryland; Johns Hopkins University School of Medicine, Baltimore, Maryland; Johns Hopkins Bayview Medical Center, Forest Hill, Maryland; Johns Hopkins University School of Medicine, Baltimore, Maryland; Johns Hopkins Bayview Medical Center, Baltimore, Maryland; Johns Hopkins Adult Burn Center, Baltimore, Maryland; Johns Hopkins University School of Medicine, Baltimore, Maryland; Johns Hopkins University School of Medicine, Baltimore, Maryland; Johns Hopkins Bayview Medical Center, Forest Hill, Maryland; Johns Hopkins University School of Medicine, Baltimore, Maryland; Johns Hopkins Bayview Medical Center, Baltimore, Maryland; Johns Hopkins Adult Burn Center, Baltimore, Maryland; Johns Hopkins University School of Medicine, Baltimore, Maryland; Johns Hopkins University School of Medicine, Baltimore, Maryland; Johns Hopkins Bayview Medical Center, Forest Hill, Maryland; Johns Hopkins University School of Medicine, Baltimore, Maryland; Johns Hopkins Bayview Medical Center, Baltimore, Maryland; Johns Hopkins Adult Burn Center, Baltimore, Maryland; Johns Hopkins University School of Medicine, Baltimore, Maryland; Johns Hopkins University School of Medicine, Baltimore, Maryland; Johns Hopkins Bayview Medical Center, Forest Hill, Maryland; Johns Hopkins University School of Medicine, Baltimore, Maryland; Johns Hopkins Bayview Medical Center, Baltimore, Maryland; Johns Hopkins Adult Burn Center, Baltimore, Maryland; Johns Hopkins University School of Medicine, Baltimore, Maryland; Johns Hopkins University School of Medicine, Baltimore, Maryland; Johns Hopkins Bayview Medical Center, Forest Hill, Maryland; Johns Hopkins University School of Medicine, Baltimore, Maryland; Johns Hopkins Bayview Medical Center, Baltimore, Maryland; Johns Hopkins Adult Burn Center, Baltimore, Maryland; Johns Hopkins University School of Medicine, Baltimore, Maryland; Johns Hopkins University School of Medicine, Baltimore, Maryland

## Abstract

**Introduction:**

The complex nature of burn pain has debilitating effects on burn patients’ physiological and psychological wellbeing. Characterized by its overwhelming intensity and extensive duration, burn pain involves inflammatory and neuropathic components. These pain responses vary in depth, severity, and sensation during and after the healing process. Despite best efforts, burn pain remains a widespread challenge for providers to effectively predict and address.

**Methods:**

A retrospective chart review of 442 patients admitted to the Burn Center for treatment of burn injuries between January 2015 and February 2022 was conducted. Charts of patients age >18 and length of stay >4 days were included in the analysis. Mean age on admission was 50.13±17 years and 34% of the sample were female. Data on clinical and demographic factors was extracted electronically and manually from patients’ electronic medical records. Numerical pain scale ratings documented by nursing were averaged for the first and last 48 hours of patients’ hospital stay. Linear regression analysis was performed to assess significant predictors of pain prior to discharge.

**Results:**

We controlled for TBSA, length of stay, gender and psychiatric diagnosis and discovered that pain within the first 48 hours of admission and age were statistically significant predictors of average pain prior to discharge. Specifically, younger age was associated with increased pain ratings. When comparing average pain levels between the first and last 48 hours, 22% reported an increase of more than 1 point in their pain, 42% had no difference in average pain ratings, and 36% reported a decrease of more than 1 point. Prior to discharge, 36% of the sample reported pain higher than 6 and 17% reported pain greater than 7.

**Conclusions:**

Heightened pain is challenging in burn injuries even prior to discharge, especially for younger patients and those who report initial high levels of pain. For many burn survivors, pain remains the same or worsens from admission to discharge, putting them at risk for negative outcomes such as chronic pain, PTSD, suicidality, sleep disturbances, and reduced function. Future research is needed to determine if early intervention can serve to mitigate these risks and improve long term recovery and quality of life.

**Applicability of Research to Practice:**

Patients at risk for increased pain upon discharge can be identified by understanding the factors contributing to this phenomenon during early treatment. Early, targeted, evidence-based interventions during treatment and following discharge will allow effective management of pain. Younger patients and patients with higher initial pain should be closely monitored and given multimodal pain interventions, thereby enhancing their comfort and overall recovery process.

